# Molecular characteristics of carbapenem-resistant gram-negative bacilli in pediatric patients in China

**DOI:** 10.1186/s12866-023-02875-0

**Published:** 2023-05-18

**Authors:** Lijun Yin, Lu Lu, Leiyan He, Guoping Lu, Yun Cao, Laishuan Wang, Xiaowen Zhai, Chuanqing Wang

**Affiliations:** 1 Department of Nosocomial Infection Control, Children’s Hospital of Fudan University, Shanghai, China; 2 The Clinical Microbiology Laboratory, Children’s Hospital of Fudan University, Shanghai, China; 3 Department of Pediatric Intensive Care Unit, Children’s Hospital of Fudan University, Shanghai, China; 4 Department of neonatal intensive care unit, Children’s Hospital of Fudan University, Shanghai, China; 5 Department of Neonatal room, Children’s Hospital of Fudan University, Shanghai, China; 6 Department of Hematology, Children’s Hospital of Fudan University, Shanghai, China; 7 Department of Nosocomial Infection Control and the Clinical Microbiology Laboratory, Children’s Hospital of Fudan University, Shanghai, China

**Keywords:** Carbapenem-resistant *K. pneumonia*e, Carbapenem-resistant *A. baumannii*, Carbapenem-resistant *P. aeruginosa*, KPC-2, NDM-1, OXA-23

## Abstract

**Background:**

Carbapenem-resistant gram-negative bacilli (CR-GNB) have been increasingly reported in China. However, dynamic monitoring data on molecular epidemiology of CR-GNB are limited in pediatric patients.

**Results:**

300 CR-GNB isolates (200 Carbapenem-resistant *K. pneumonia*e (CRKP), 50 carbapenem-resistant *A.baumannii* (CRAB) and 50 carbapenem-resistant *P. aeruginosa* (CRPA)) were investigated. The predominant carbapenemase gene was *bla*_NDM−1_ (73%) and *bla*_KPC−2_ (65%) in neonates and non-neonates. Meanwhile, the predominant STs were ST11 (54%) in neonates and ST17 (27.0%) and ST278 (20.0%) in non-neonates. Notably, a shift in the dominant sequence type of CRKP infections from ST17 /ST278-NDM-1 to ST11-KPC-2 was observed during the years 2017–2021 and KPC-KP showed relatively higher resistance to aminoglycosides and quinolones than NDM-KP.*Bla*_OXA−23_ was isolated from all the CRAB isolates while only one isolate expressing *bla*_BIC_ and 2 isolates expressing *bla*_VIM−2_ were found in CRPA isolates. ST195 (22.0%) and ST244 (24.0%) were the most common in CRAB and CRPA isolates and all the STs of CRAB belonged to CC92 while CRPA presents ST types with diversity distribution.

**Conclusion:**

CRKP showed different molecular phenotypes in neonates and non-neonates and was changing dynamically and high-risk clone of ST11 KPC-KP should be paid more attention. Most CRKP and CRAB strains shared the same CCs, suggesting that intrahospital transmission may occur, and large-scale screening and more effective measures are urgently needed.

## Background

Carbapenem-resistant Gram-negative bacilli (CR-GNB), namely, carbapenem-resistant Enterobacteriaceae (CRE), carbapenem-resistant *Acinetobacter baumannii* (CRAB), and carbapenem-resistant *Pseudomonas aeruginosa* (CRPA), are a serious cause of health care-associated infections and an emerging health threat worldwide given the scarcity of treatment options, high mortality rates and hospital costs and ultimately increase the difficulty of nosocomial infection prevention and control (IPC) [1]. At present, infections caused by CR-GNB are on the rise worldwide [2]. In parallel to what has been observed in adult populations, a progressive increase in the incidence of infections due to multidrug-resistant microorganisms has occurred in children. Published studies during the last decade have reported that the frequency of carbapenem resistance in children in the United States increased from 0% to 1999 and 2000 to 0.47% in 2010 and 2011 among Enterobacterales isolates [3], from 9.4% to 1999 to 20% in 2012 among *P. aeruginosa* isolates [4], and from 0.6% to 1999 to 6.1% in 2012 among *A. baumannii* isolates [5].According to the CHINET surveillance of bacterial resistance in China in 2018 [6], a remarkable increase in resistance to imipenem and meropenem was also seen in *K. pneumoniae* from 3.0 to 25.0% and from 2.9 to 26.3% during the period from 2005 to 2018. The drug resistance rates of *P. aeruginosa* and *A. baumannii* to carbapenems were above 30% and 70%, respectively. CR-GNB strains have the potential for widespread transmission of resistance via mobile genetic elements [7], which can lead to significant outbreaks [8]. Hence, understanding its drug resistance mechanism and molecular characteristics is a research priority.

Therefore, in present study we investigated the antibiotic resistance characters, as well as the carbapenemase genes, multilocus sequence typing (MLST) of CR-GNB in the pediatric patients. This is the first report about the epidemiology of pediatric CR-GNB isolates for five years from 2017 to 2021 in China, our study will be very essential to the control and clinical administration of pediatric CR-GNB infections.

## Methods

### Study design and definition

The Children’s Hospital of Fudan University is an 800-bed tertiary-care teaching hospital in Shanghai, China. The hospital has an average of 50 000 admissions per year, among whom 75% came from other provinces or cities in China.

300 non-repetitive isolates from non-repetitive patients (200 Carbapenem-resistant *K. pneumonia*e (CRKP), 40 per year, including 100 neonatal and 100 non-neonatal, 50 CRAB and 50 CRPA) from 2017 to 2021 were selected for antimicrobial susceptibility testing, carbapenem resistance gene and MLST typing.

CRE were defined as the Enterobacteriaceae strains which presented resistance to either of ertapenem, imipenem or meropenem; CRAB and CRPA were defined as the *A. baumannii* or *P. aeruginosa* strains which presented resistance to either imipenem or meropenem in accordance with the breakpoints of CLSI guidelines [9].The CRKP,CRAB and CRPA strains from 2017 to 2021 were selected by random method, and the first qualified strain isolated from each patient after their first admission was included in the study. All isolates were stored at -80 °C, sub-cultured aerobically at 35 ± 2 °C and transferred twice prior to testing.CR-GNB strains collected from the upper respiratory or rectal site were considered as colonization and excluded from this study.

### Antimicrobial susceptibility testing

Routine CR-GNB infection surveillance was analyzed using culture and antimicrobial susceptibility tests. Strains were identified by MALDI-TOF biotyper mass spectrometry (Bruker Company, Germany). For CRAB strains, further confirmation is carried out to detect *bla*_OXA−51_ using molecular methods. Antimicrobial susceptibility tests (ASTs) were performed by automatic Vitek2 compact machines. The standard strains *Escherichia coli* ATCC25922 and *Escherichia coli* ATCC35218 (enzyme-producing strains) were used as quality control strains for antimicrobial susceptibility tests. *Escherichia coli* ATCC 25,922 and *Pseudomonas aeruginosa* ATCC 27,853 were used as quality control strains for the drug sensitivity test by the disk diffusion method. Clinical information for CR-GNB-positive patients was systematically reviewed from electronic medical records.

### Molecular detection of resistance genes

Carbapenemase (Class A carbapenemase: *bla*_KPC_, *bla*_GES_, *bla*_SME_, *bla*_IMI_; Class B carbapenemase *bla*_NDM_, *bla*_GIM_, *bla*_BIC_, *bla*_SIM_, *bla*_DIM_, *bla*_IMP_, *bla*_SPM_, *bla*_AIM_, *bla*_VIM_, *bla*_DHA_; Class D carbapenemase: *bla*_OXA−23_, *bla*_OXA−24_, *bla*_OXA−48_, *bla*_OXA−51_, *bla*_OXA−58_, *bla*_OXA−143_) genes were investigated by polymerase chain reaction (PCR) with previously described primers [10, 11]. PCR amplicons were sequenced, and the resulting DNA sequences were compared with those available in the NCBI GenBank database using BLAST searches.

### MLST

MLST was carried out according to protocols available at the MLST Pasteur website (https://bigsdb.pasteur.fr/index.html) and MLST PubMed website (https://pubmlst.org/). Clonal complexes (CCs) were defined as different by one or two alleles. A minimum spanning tree of CRKP, CRAB and CRPA positive isolates were constructed with the BioNumerics software.

### Statistical analysis

All data are expressed as rates for categorical variables. Comparison between groups were performed by using chi-square test for categorical variables; p < 0.05 was regarded as statistically significant. All statistical analyses were performed with the statistical software SPSS 21.0.

## Results

### Clinical characteristics of patients with CR-GNB

From 2017 to 2021, 5078 CR-GNB-positive patients were found, with CRE, CRAB and CRPA patients accounted for 58.1% (2590), 26.6% (1350) and 15.3% (778), respectively. CRKP (74.1%, 2186) was the most frequently isolated pathogen in CRE patients. Other CRE isolates included *Escherichia coli* (9.6%, 283), *Enterobacter cloacae* (7.1%, 209), *Klebsiella aerogenes* (2.4%, 71), *Klebsiella acidogenes* (2.1%, 62), *Klebsiella cepacia* (0.8%, 24) and other (3.9%, 115).

CPKP isolates were primarily collected from lower respiratory (54.0%), followed by urine (33.0%), blood (8.0%) and others (5.0%), and mainly from the newborn room (20.5%), NICU (28.5%) and PICU (19.0%). CRAB and CRPA were also primarily collected from lower respiratory (82.0% and 77.9%), and mainly from the PICU (74.0% and 75.9%).

### Carbapenemase genes of CR-GNB isolates

**Fig. 1:**
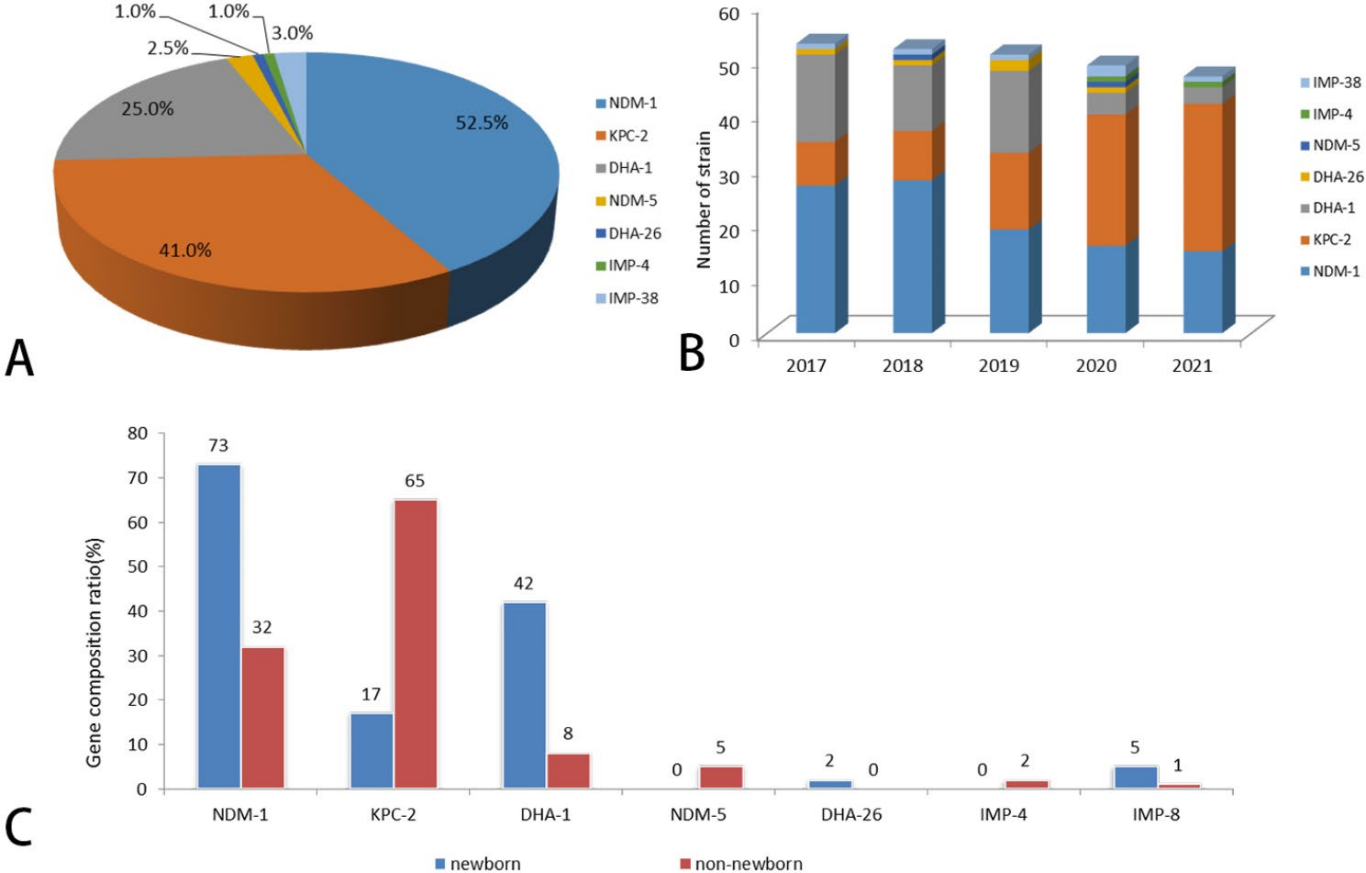
Carbapenemase genes of Carbapenem-resistant *K. pneumonia*e (CRKP) strains

Seven kinds of resistance genes were detected in the 200 CRKP isolates, of which *bla*_NDM−1_ (52.5%,105) was the most common, followed by *bla*_KPC−2_ (41.0%,82), *bla*_DHA−1_ (25.0%,50), *bla*_IMP−38_ (3.0%,6), *bla*_NDM−5_ (2.5%,5), *bla*_DHA−26_ (1.0%,2), and *bla*_IMP−4_ (1.0%,1) (Fig. 1A). It is worth noting that NDM-1 was predominant in 2017 and 2018, whereas KPC-2 became the predominant gene from 2019 (Fig. 1B). Meanwhile, neonatal and non-neonatal CRKP isolates showed different molecular characteristics (Fig. 1C); the predominant carbapenemase gene was *bla*_NDM−1_ (73%) in neonates, followed by *bla*_DHA−1_ (42.0%), *bla*_KPC−2_ (17.0%), *bla*_IMP−8_ (5.0%), and *bla*_DHA−26_ (2.0%). The predominant carbapenemase gene was *bla*_KPC−2_ (65%) in non-neonatal patients, followed by *bla*_NDM−1_ (32%), *bla*_DHA−1_ (8%), *bla*_NDM−5_ (5.0%), *bla*_IMP−4_ (2.0%) and *bla*_IMP−8_ (1.0%). Besides, these CRKP strains were also found to contain 8 different profiles of combination expression (29.0%, 58/200) of carbapenemases and the majority of isolates (70.7%,41/58) coharboured carbapenemase genes *bla*_NDM−1_ and *bla*_DHA−1_,followed by 7 isolates (12.1%) coharboured carbapenemase genes *bla*_NDM−1_ and *bla*_KPC−2_.

The carbapenemase genes *bla*_OXA−23_ and *bla*_OXA−51_ were found in all the 50 CRAB isolates while only one isolate expressing *bla*_BIC_ and 2 isolates expressing *bla*_VIM−2_ were found in CRPA isolates.

A Proportion of carbapenemase genes of CRKP isolates; B Number of carbapenemase genes of CRKP isolates between 2017 and 2021; C Comparison of carbapenemase genes composition rate of CRKP isolates between newborn and non-newborn.

### MLST of CR-GNB isolates

**Fig. 2:**
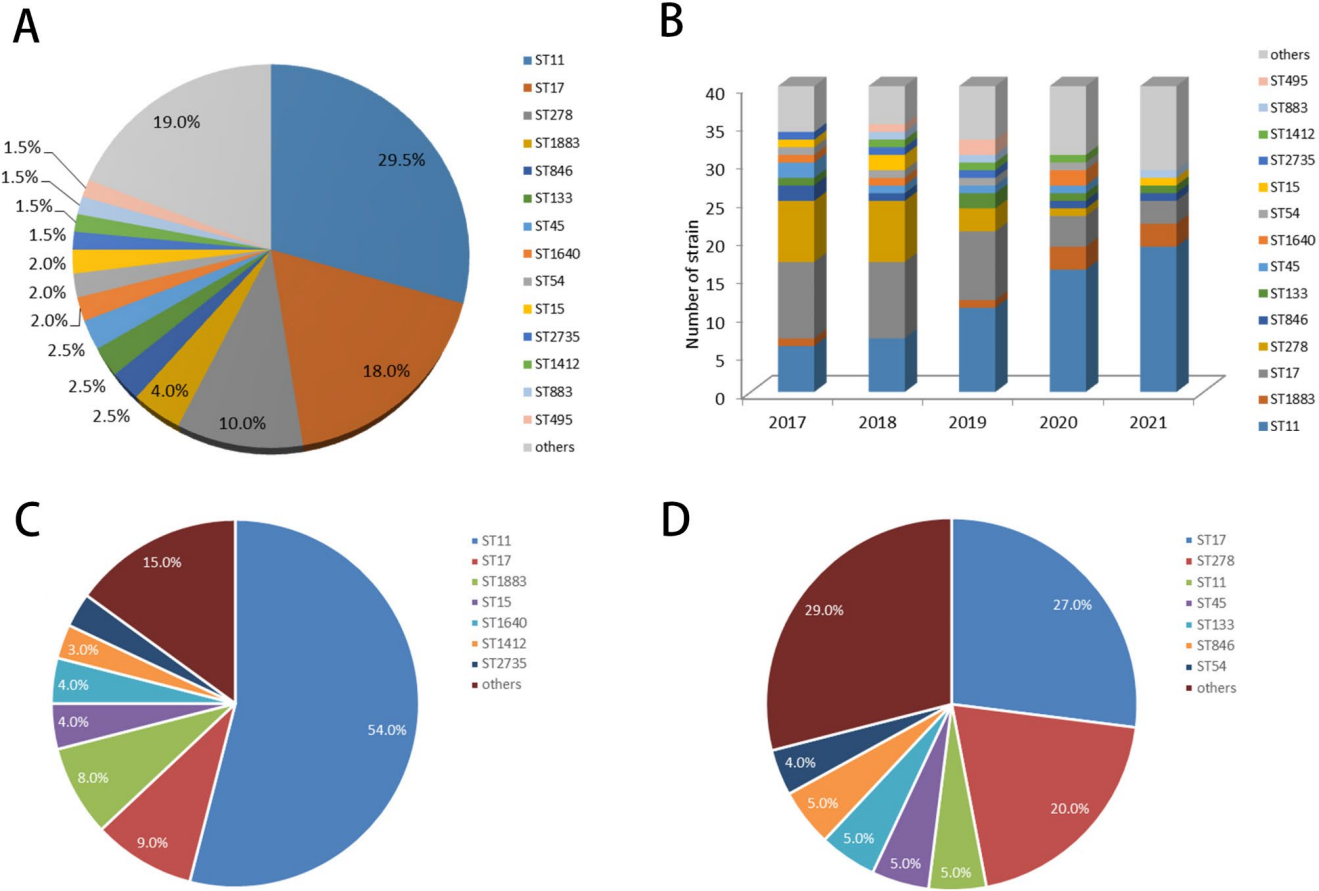
**Multilocus sequence typing (MLST) of Carbapenem-resistant*K. pneumonia*e (CRKP) strains** A, Proportion of MLST of CRKP isolates; B, Number of MLST of CRKP isolates between 2017 and 2021; C, Proportion of MLST of CRKP isolates in newborn (C) and non-newborn (D)

**Fig. 3:**
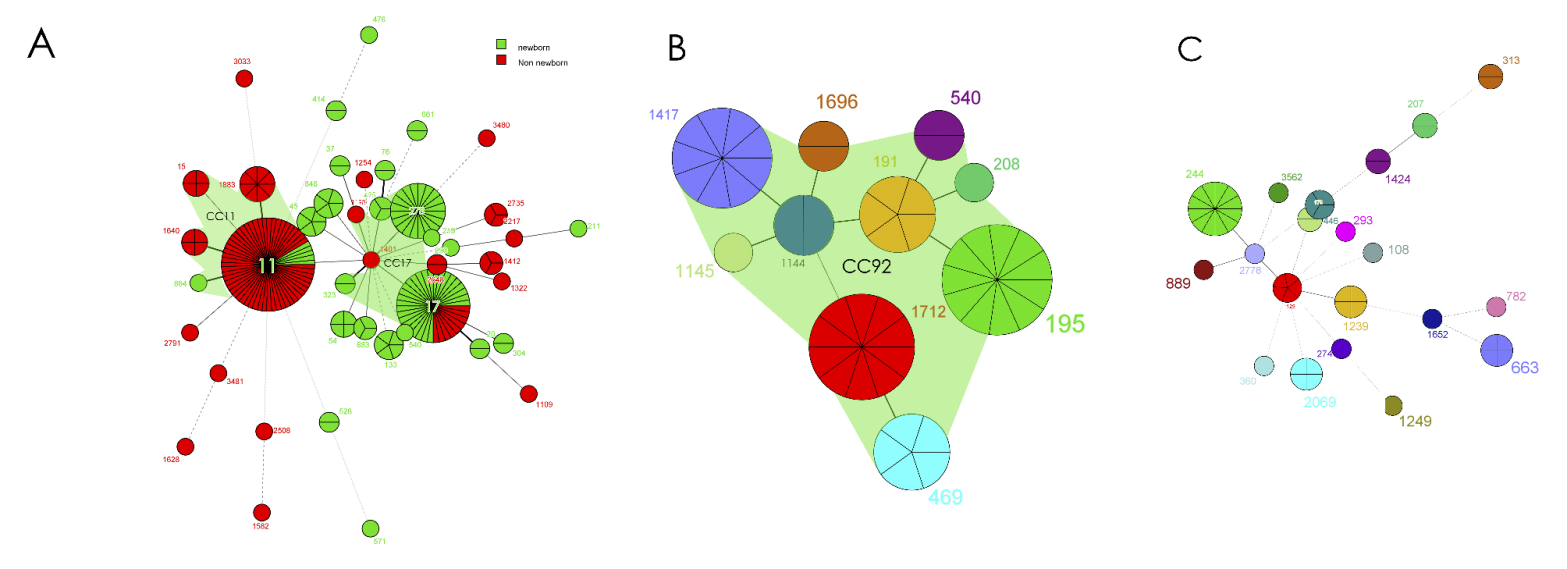
**Minimum spanning tree of Multilocus sequence typing (MLST) created by BioNumerics.** Each solid circle denotes one ST, and the area of the circle is proportional to the number of isolates. The lines connecting the circles indicate the relationship between different STs. Different types of lines represent a difference in one allele (solid lines), two alleles (dashed lines) and three or more alleles (dotted lines). Clonal complexes (CCs) were defined as different by one/two alleles. The coloured zones surrounding the STs indicate that they belong to the same. STs enclosed by shaded areas constitute a clonal complex (CC). Of the CRKP STs, ST11 and ST1883, ST1640, ST15, ST864 belonged to CC11, ST17 and ST278, ST1401, ST495, ST1198 belonged to CC17. All the CRAB STs belonged to CC92.

43 STs were found in CRKP isolates, of which ST11 (29.5%) was the most common, followed by ST17 (18.0%) and ST278 (10.0%) (Fig. 2A). It is worth noting that ST17 and ST278 were predominant in 2017 and 2018, whereas KPC-2 became the predominant ST types from 2019 (Fig. 2B). Meanwhile, the predominant STs were ST11 (54%) in neonates (Fig. 2C) and ST17 (27.0%) and ST278 (20.0%) in non-neonates (Fig. 2D). A total of 94.9% (56/59) of ST11 expressed *bla*_KPC−2,_ while 98.2% (55/56) of ST17 and ST278 expressed *bla*_NDM−1_.ST11 and ST1883, ST1640, ST15, ST864 belonged to CC11, ST17 and ST278, ST1401, ST495, ST1198 belonged to CC17(Fig. 3A). 38.0% (76/200) and 31.5% (63/200) of CRKP belonged to CC11 and CC17.

Ten STs were found in CRAB isolates, with ST195 (22.0%) being the most common, followed by ST1712 (20.0%) and ST1417 (18.0%). All the STs of CRAB belonged to CC92 (Fig. 3B). Twenty STs were found in CRPA isolates, with ST244 (24.0%) being the most common, followed by ST1129 (10.0%) (Fig. 3C).

Their evolutionary relationships are shown in Fig. 3.

**A**, CRKP,carbapenem-resistant Klebsiella pneumonia; **B**, CRAB,carbapenem-resistant Acinetobacter baumannii;**C**, CRPA,carbapenem-resistant Pseudomonas aeruginosa.

### Antimicrobial-resistant characteristics of CR-GNB strains

**Fig. 4:**
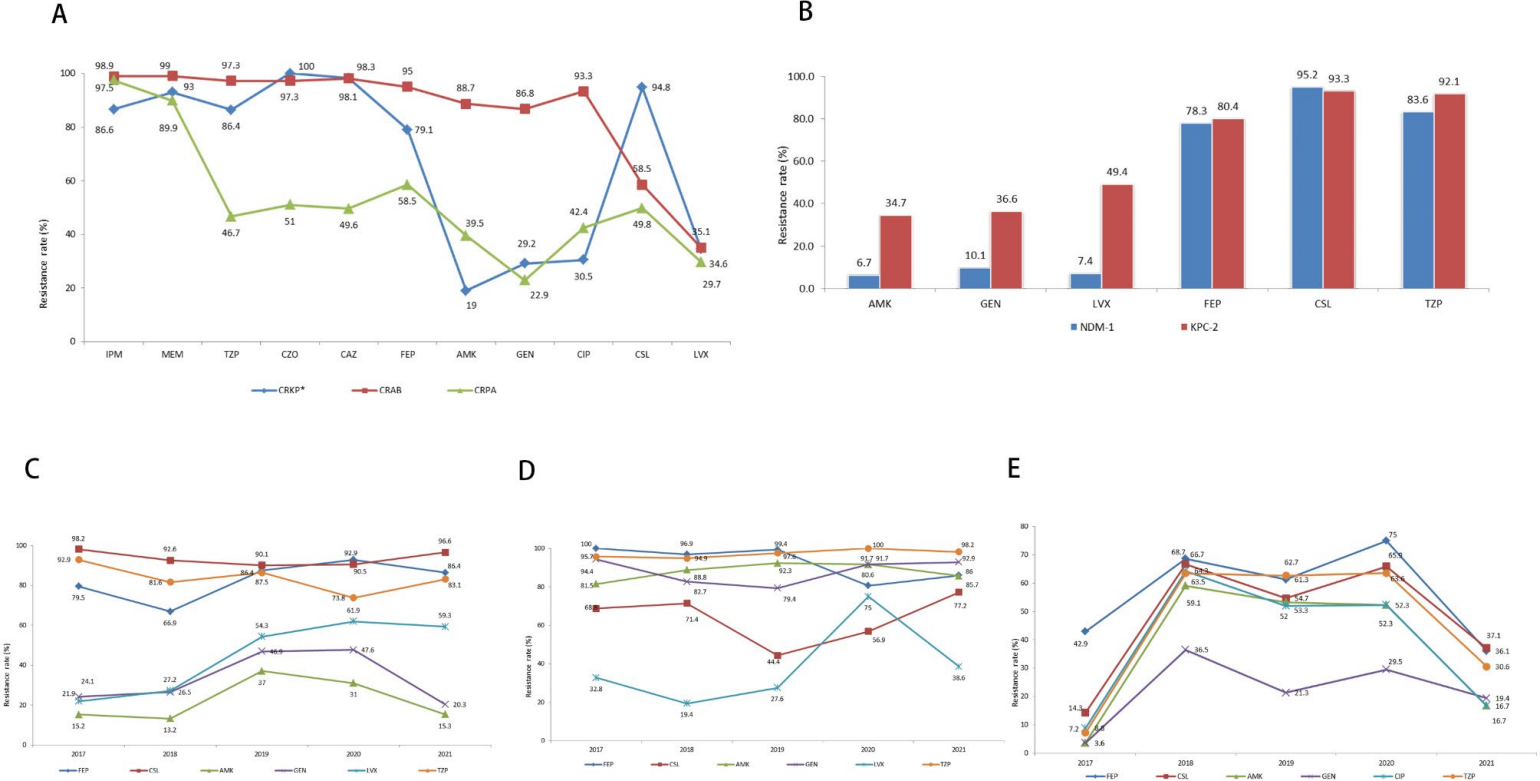
The resistance rate of Carbapenem-resistant gram-negative bacilli isolates (%)

CRKP, CRAB and CRPA all maintained high resistance rate to carbapenems (> 90%). For other antibacterial drugs see Fig. 4A. Except for levofloxacin, CRAB had higher resistance to aminoglycosides and quinolones than CRKP and CRPA.CRKP and CRAB strains were resistant to almost all first- and third-generation cephalosporin and enzyme inhibitors, while CRPA was relatively low. The resistance rates to cefoperazone/sulbactam varied across different CR-GNB strains, where the CRKP strains showed higher resistance than CRAB and CRPA (Fig. 4A). Resistance rate of the KPC-2 and NDM-1 CRKP isolates were further analyzed and results found that KPC-2 showed relatively higher resistance to amikacin,gentamicin and levofloxacin (Fig. 4B).

CRKP, CRAB and CRPA showed different drug resistance trend from 2017 to 2021. For CRKP, the resistance rate to cefepime, cefoperazone/sulbactam and piperacillin/tazobactam maintained high level. The resistance rate to amikacin, gentamicin and levofloxacin displayed an upward trend from 2017 to 2020 while it decreased in 2021(Fig. 4C).

For CRAB, except cefepime and piperacillin/tazobactam, the resistance rate to amikacin and gentamicin also maintained high level. The resistance rate to levofloxacin displayed a similar upward trend with CRKP from 2017 to 2020 while it decreased in 2021. The resistance rate to cefoperazone/sulbactam showed a downward trend from 2017 to 2019 but it increased from 2020 (Fig. 4D).

For CRPA, resistance rate to gentamicin was the lowest and the resistance rate to all the drugs showed similar trends, with significant increase in 2018, slight fluctuation in 2018–2020, and significant decrease in 2021(Fig. 4E).

Comparison of resistance rate in Carbapenem-resistant *Klebsiella pneumonia* (CRKP), Carbapenem-resistant *Acinetobacter baumannii* (CRAB) and Carbapenem-resistant *Pseudomonas aeruginosa***(**CRPA) isolates (A), KPC-2 and NDM-1 of CRKP isolates (B), drug resistance trend from 2017 to 2021 in CRKP(C),CRAP(D) and CRPA(E) isolates.

*The resistance rate of CRKP isolates was 100% to cefuroxime, ceftriaxone, cefotaxime, amoxicillin/clavulanic acid and ampicillin, 96.4%, 91.6%, 85.1%, 62.3%, 46.5%, 25.7% and 17.7% to cefoxitin, aztreonam, cefmetazole, cefotetan, nitrofurantoin, trimethoprim/sulfamethoxazole and tobramycin.

IPM,imipenem;MEM,meropenem;TZP, piperacillin/tazobactam;CZO,Cefazolin;CAZ, Ceftazidime; FEP, cefepime; AMK,amikacin;GEN,gentamicin;CIP, ciprofloxacin;CSL, cefoperazone/Sulbactam;LVX, levofloxacin;

## Discussion

Molecular research found a high clonal diversity among CRKP isolates, with the predominance of the ST11 strain expressing KPC-2 in non-neonates and the ST17/ST278 strain expressing NDM-1 in neonates and a shift in the dominant sequence type of CRKP infections from ST17 /ST278-NDM-1 to ST11-KPC-2 was observed during the years 2017–2021. This study revealed that *bla*_OXA−23_-like is the major mechanism responsible for the carbapenem resistance phenotype for CRAB while only one isolate expressed *bla*_BIC_ and two isolates expressed *bla*_VIM−2_ were found in the CRPA isolates.

CR-GNB has become a major concern worldwide [12]. Many factors, including the ease of international travel for medical tourism and migration, and the importation of food products, have been responsible for introducing these microorganisms to several countries far beyond their country of origin [13, 14]. *Bla*_NDM#x2212;1_ is the main reported CRKP gene in neonatal patients [15], while *bla*_KPC−2_ has been reported in adults and older children [8, 10]. In this study, we also found that NDM-KPN is more prevalent in neonates (73%) while KPC-KPN is mainly detected from non-neonates (65%), indicating the different molecular and epidemiological characteristics of CPKP between different ages. Additionally, we found two clonal complexes among CRKP strains, with the predominance of CC11 expressing KPC-2 in non-neonates and CC17 expressing NDM-1 in neonates. Importantly the dominant sequence type and carbapenemase genes was changing during the years 2017–2021, indicating the importance of dynamic monitoring and more effective control measures of infection because the shift of resistance genes and ST types may lead to the more severe drug-resistance results. Compared with other carbapenem genes, *bla*_KPC_ showed relatively higher resistance to amikacin, gentamicin and levofloxacin in this study. Other studies also found that *bla*_KPC_ shows stronger virulence and transmission performance, with several hospital outbreaks (most often due to KPN with *bla*_KPC−2_) reported in adults [8, 16]. Meanwhile, most of the ST11 CRKP expressed *bla*_KPC−2_ and the pandemic of ST11 KPC-KPN is associated with the horizontal gene transfer mediated by transposons or plasmids [17], so we must be concerned about the potential spread of the high-risk clones of ST11 KPC-KPN among children in China.

CRAB is ranked first among gram-negative bacteria in the WHO priority list of antibiotic-resistant bacteria, followed by CRPA [18]. *A. baumannii* is a nosocomial pathogen of clinical significance, which has been implicated in a wide spectrum of hospital acquired infections, mostly among immunocompromised patients in intensive care units. The main reason for CRAB in children was the strains carrying *bla*_OXA-23_ in our study, which was consistent with the results at home and abroad from adult patients and hospital environments, suggesting that *bla*_OXA-23_-carrying CRAB has become endemic [19–22]. Meanwhile, all the CRAB STs belonged to CC92, suggesting that clonal spread of CRAB CC92 isolates longitudinally is the possible reason for carbapenem resistance rate increase and correlate with high level carbapenem resistance in our hospital.CC92 is a widespread variant that has advantages with respect to acquiring resistance determinants and surviving in the nosocomial environment, which makes it preferentially selected under antibiotic pressure [20]. However, one isolate expressed *bla*_BIC_, and two isolates expressing *bla*_VIM-2_ were found in CRPA isolates. CRPA is usually multifactorial and can be caused by several different mechanisms [23], including acquire resistance to carbapenems by acquisition of transferable genes encoding carbapenemases, repression or inactivation of the carbapenem porin OprD and hyperexpression of the chromosomal cephalosporinase AmpC [23],so further research is needed. Meanwhile, a large proportion of the CRPA patients were infected by different STs in our study and ST244 was the major ST, which was similar to research in China [24].

Antimicrobial treatment must be individualized according to the severity and the source of infection and the susceptibility profile of the isolated bacteria. CRAB showed more severe form of drug resistance than CRKP and CRPA though they showed different drug resistance trend from 2017 to 2021. The prevalence of COVID-19 has crashed antibiotic stewardship and increased the global usage of antibiotics and personal protective equipment, which may have played role in the changes of carbapenem resistance phenotype, ST types and drug resistance trends in present study. Combination therapy are recommended in adults and the antibiotics most frequently used in combination therapy, in descending order, were colistin (n = 63), aminoglycosides (n = 46), carbapenems (n = 30), tigecycline (n = 26), aztreonam (n = 2), and tetracyclines (n = 2) [2].Unfortunately, specific recommendations for antibiotic therapy for CR-GNB infections in pediatric patients are limited and based on studies including only adults [25]. Limited experience with those drugs makes them less recommendable in children [26].

Our study has limitations. First, as important therapeutic drugs [8], our data on the drug sensitivity of ceftazidime/avibactam, polymixin B and tigecycline are limited. Besides, the comparative analysis of resistance genes and ST of CRAB and CRPA types between years were not conducted because the single clone group of CRAB and limited positive strains of CRPA. Finally, we did not distinguish whether the isolated bacteria were colonized or clinically infected and link these data to clinical dissemination of clone types, so further research is needed in future research.

## Conclusions

In conclusion, CRKP showed different molecular phenotypes in neonates and non-neonates and is changing dynamically, providing valuable information for the clinical management of CRKP in pediatric patients. CRAB strains shared the same CCs, suggesting that intrahospital transmission may occur, and large-scale screening and more effective measures are urgently needed. Our study provides the stepstone for the future expanded research associated with multicenter and further resistant mechanism surveillance to prevent further possible dissemination in this region.

## Data Availability

The datasets used and/or analysed during the current study are available from the corresponding author on reasonable request.
